# Investigating the hydration of C3A in the presence of the potentially toxic element chromium–a route to remediation?†

**DOI:** 10.1039/d2ra04497h

**Published:** 2022-10-12

**Authors:** Rebecca Rae, Margaret C. Graham, Caroline A. Kirk

**Affiliations:** EaStCHEM School of Chemistry, University of Edinburgh Joseph Black Building, David Brewster Road Edinburgh EH9 3FJ Scotland UK Caroline.Kirk@ed.ac.uk +44 (0)131 650 4840; School of Geosciences, University of Edinburgh Edinburgh EH9 3JN Scotland UK

## Abstract

Pollution by hexavalent chromium is a growing, global problem. Its presence in public water systems is often the result of industrial activities, both past and present. In this study, tricalcium aluminate (C3A, Ca_3_Al_2_O_6_) is added to solutions of varying concentrations of potassium chromate (K_2_CrO_4_) and samples of both the solid and liquid are taken at various time intervals to monitor the removal of chromium from the solutions. Solution concentrations of 0.2 M, 0.1 M, 0.02 M, and 0.01 M are used, and the chromium concentration is found to reduce in all cases. For the 0.02 M solution the chromium concentration is reduced from 1040 ppm to 3.1 ppm in 1 week, and the chromium concentration of the 0.01 M solution is reduced from 520 ppm to 0.26 ppm in only one day of reaction with the C3A. The chromium removed from solution is identified in the solid products, which were fully characterised as being a mixture of ettringite (Ca_6_[Al(OH)_6_]_2_(CrO_4_)_3_·26H_2_O) and monochromate (Ca_4_[Al(OH)_6_]_2_CrO_4_·8H_2_O) phases from analysis of Powder X-ray Diffraction and Fourier Transform Infrared Spectroscopy data. The work presented here is a proof of concept study to investigate C3A as a potential material for the removal of hexavalent chromium from solution. The results from this study are initial steps towards development of this as a technology for hexavalent chromium remediation.

## Abstract 2

A calcium aluminate material has been successfully used to remove toxic hexavalent chromium from water. A thorough powder X-ray diffraction study was done to determine the composition of the products.

## Graphical abstract

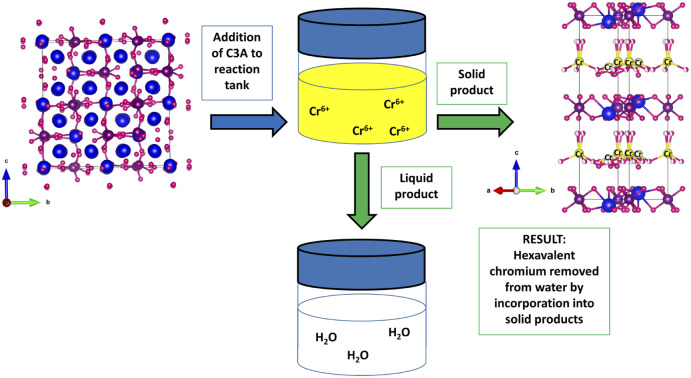

## Introduction

There is a growing global need to remediate wastewater which contains potentially toxic elements (PTEs). Some common PTEs are lead, arsenic, and chromium. The volume of industrial wastewater produced is only expected to increase as the global population increases. By 2030 the global demand for energy is expected to increase by 40% and the global demand for water is expected to increase by 50%.^1^ Alongside this, the volumes of industrial wastewater is expected to be double their 2007 values by 2025 (ref. 2) and as such, the market for industrial water treatment technologies is predicted to grow significantly.^3^

If untreated wastewater is discharged into the environment, it can affect both freshwater supplies and, with its eventual destination being the ocean, the marine ecosystem. The release of untreated wastewater can have huge consequences for society, impacting both human health, causing a reduction in global productivity, as well as animal health, influencing global agriculture and food production. There may be wider implications for the tourism and housing markets based around bodies of water that end up polluted, impacting the economy. Therefore, there are economic as well as public health incentives to effectively treat wastewater.

Along with the health, environmental, and economic benefits of treating wastewater, finding ways to reuse treated wastewater could solve a wide range of problems. Many countries are already facing water scarcity and this problem will only worsen due to the increasingly significant effects of climate change. Treated wastewater could be used for agricultural and industrial uses, reducing the need for freshwater.

Chromium, when in its hexavalent state Cr(vi), is a well-known genotoxic carcinogen, damaging DNA and causing cancer and other mutagenic damage,^4^ and as such poses a threat to human health and life.^5^ Chromium is most often present as a pollutant in wastewater as a result of untreated industrial waste runoff^6^ where it can enter the groundwater.^7,8^ The industrial activities that can result in the release of hexavalent chromium include metal (chrome) plating, textile preservation and tanning.^9^ The Polmadie Burn in Glasgow, Scotland, is a site polluted with hexavalent chromium. The pollution in this burn originated from historic industrial plants which disposed of chromite ore processing residue (COPR) to landfill. COPR contains chromium in multiple oxidation states, with Cr(vi) being more mobile and therefore readily released to the groundwater.^10,11^ This is a problem that remains to this day (2022) with efforts to remediate the waste ongoing.^12–14^

The objective of most remediation strategies for chromium contamination is to reduce soluble Cr(vi) to the less harmful and insoluble Cr(iii).^15^ Traditional environmental reductants such as Metallic Fe, Fe(ii), organic materials or reduced S-containing species have met with only limited success at high pH (>11), COPR-contaminated sites.^16–18^ Recently, studies using bismuth-based photocatalysts have been carried out and successfully reduced Cr(vi) to Cr(iii),^19,20^ with higher rates of reduction occurring at lower acidic pHs. However, remediation through a reduction process carries the risk of reoxidation of the Cr(iii) and remobilisation into the environment. To remove this risk, one option is to stabilise the toxic element through binding in a matrix, a common method used for encapsulation of radioactive waste in cement.^21^ One of the problems of stabilising waste in a cement matrix is the high pH environment of cement. By first solidifying and encapsulating the waste in cement minerals, this problem can be avoided.^22^

Tricalcium aluminate (Ca_3_Al_2_O_6_, C3A) is a phase present in cement clinkers. Its crystal structure is cubic with space group Pa3.^23^ It is composed of AlO_4_ tetrahedra which corner share to form 6-fold rings. The CaO_6_ octahedra edge share with other CaO_6_ octahedra and corner share with the AlO_4_ to form the 3D network that makes up the crystal structure (structural diagram provided in ESI†). During the cement hydration process C3A reacts with gypsum (CaSO_4_·2H_2_O), which is a source of SO_4_^2−^, to form both ettringite (Ca_6_[Al(OH)_6_]_2_(SO_4_)_3_·26H_2_O) and monosulfate (Ca_4_[Al(OH)_6_]_2_SO_4_·6H_2_O).^24,25^*In situ* neutron powder diffraction studies have been carried out by Christensen *et al.* to monitor the reactions of mixtures of C12A7 (Ca_12_Al_14_O_33_) and gypsum.^26^ They observed the initial formation of ettringite then, once all the gypsum has reacted, ettringite begins to deplete and there is the formation of a monosulfate phase.

In this study, the reaction between C3A and Cr(vi), in the form of CrO_4_^2−^ anions, is predicted to form the analogous products; chromate ettringite (Ca_6_[Al(OH)_6_]_2_(CrO_4_)_3_·26H_2_O),^27^ and monochromate (Ca_4_[Al(OH)_6_]_2_CrO_4_·6H_2_O).^28,29^ The precipitation of chromium-containing ettringite and monosulfate analogue phases will result in the concentration of chromium in a solution being reduced, with potential full remediation of the wastewater.

Understanding the remediation process is key for planning waste removal strategies. As such, lab-based studies, such as this one, are an essential first step, as these allow control of the process and the mechanisms can be understood fully before scaling up the process and testing on samples collected from polluted sources. This study aims to test a new potential chemical precipitation remediation method where the addition of C3A to Cr-containing solutions will result in the precipitation of solid phases with the chromium encapsulated. This study aims to prove the viability of using C3A to remove Cr(vi) from waste solutions. Future work would be required to develop this into a working remediation method. This study will include comprehensive characterisation of all products, solid and liquid. These results will also enhance the literature that exists in the fields of cement materials and solid state chemistry.

## Experimental methods

### Synthesis of tricalcium aluminate

Tricalcium aluminate (Ca_3_Al_2_O_6_, C3A) was prepared by grinding calcium carbonate (CaCO_3_, Acros Organics, 99%) and aluminium oxide (Al_2_O_3_, Acros Organics, 99.7%) together in a mortar and pestle, transferring to an alumina boat and firing in a furnace at 800 °C for 12 hours. The sample was then reground before firing in a furnace at 1300 °C for 48 hours. This grinding–heating process was repeated until the product (C3A) was obtained and determined to be phase-pure by Powder X-ray Diffraction (PXRD), details below.

### Addition to solution

Prepared C3A (0.25 g) was added to solutions (25 cm^3^) of potassium chromate (K_2_CrO_4_) in ultra-pure deionised water. Concentrations of 0.2 M, 0.1 M, 0.02 M, and 0.01 M were used to give a range from high solution concentration to lower and closer to environmental concentrations. The resulting mixtures were agitated on a roller mixer in HDPE bottles. The agitation time was varied and after the allotted time periods the solid products were separated from the liquid samples by vacuum filtration. The agitation times for each experiment are shown in Table 1:

**Table 1:** Agitation times at which samples were taken for various solution concentration experiments

Experiment solution concentration (M)	Agitation time (h)
0.2	1, 3, 6, 24, 168, 744
0.1	1, 3, 6, 24, 168, 744
0.02	1, 3, 6, 24, 72, 168, 744
0.01	24, 72, 120, 168

The agitation times were chosen in order to track the evolution of the phases as the reaction between the C3A and the solution progresses. The maximum time was chosen to be 1 month, or until there was evidence that all the chromium had been removed from solution.

### Characterisation of solid samples

All solid samples were characterised using PXRD, Fourier Transform Infrared Spectroscopy (FTIR), and Inductively Coupled Plasma-Optical Emission Spectrometry (ICP-OES). PXRD analysis: the samples from the 0.2 M concentration solution experiments were analysed on the High Resolution Powder X-ray Diffraction beamline (I11) at the Diamond Light Source.^30^ These synchrotron powder X-ray diffraction data were collected at room temperature using a Si-calibrated wavelength of *λ* = 0.82661 Å. The 0.1 M, 0.02 M, and 0.01 M experiment samples were analysed, at room temperature, using a D8-Advance powder X-ray diffractometer, in transmission geometry with Cu Kα_1_ radiation (*λ* = 1.5406 Å). FTIR analysis: data were collected using a PerkinElmer Spectrum Two, with an ATR (Attenuated Total Reflectance) attachment. ICP-OES analysis: a fraction of the solid product (0.01 g) was dissolved in 70% A.R. grade nitric acid (HNO_3_, 5 cm^3^) in a volumetric flask. These were then diluted 25× using 2% v/v A.R. grade HNO_3_ before ICP-OES measurements were made using a PerkinElmer Optima 8300 DV employing an RF forward power of (1500 W), with argon gas flows of 10, 0.2, and 0.6 L min^−1^, for plasma, auxiliary, and nebuliser flows, respectively. Three replicate measurements per sample were employed and a range of calibration standards for each element were prepared. The wavelengths for each element that were selected for analysis were as follows: Al-308.215 nm, Ca-317.933 nm, Cr-205.560 nm, and S-181.975 nm. For all calibration lines, the correlation coefficients for either the linear or weighted linear regressions were 0.99814 or better.

### Characterisation of liquid samples

The liquid samples were analysed using ICP-OES. These samples were diluted 20× using 2% v/v A.R grade HNO_3_ before ICP-OES measurements were made, using the same wavelengths and standards as for the solid samples.

## Results and discussion

### Analysis of the liquid samples

**Fig. 1:**
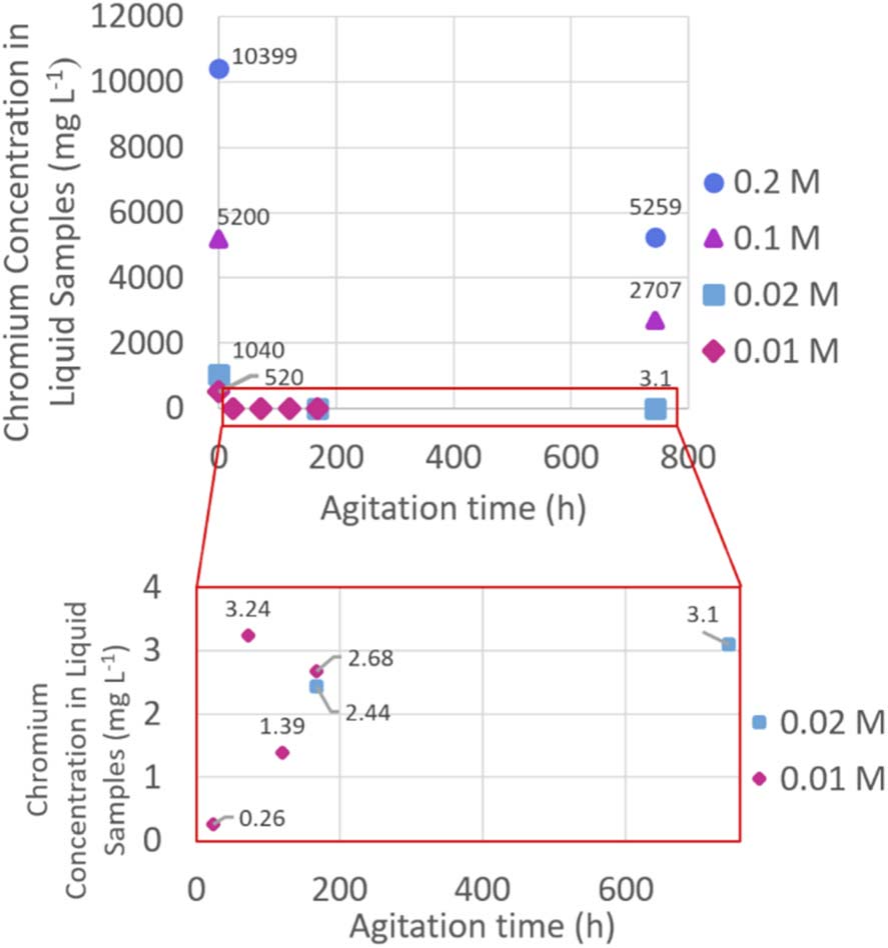
Chromium concentrations (in mg L^−1^, ppm) of the liquid samples after solid products separated, measured using ICP-OES. Low concentration region of the graph highlighted in red box.

The concentration of chromium in the liquid samples was measured using ICP-OES and this allowed calculation of how much chromium had been removed from the liquid phase after reaction with C3A at various time points (Fig. 1).

The ICP-OES results show that in all cases, significant amounts of chromium have been removed from the liquid phase. 250 mg of C3A allows 144.549 mg of chromium to be removed. With the solution volume being 25 cm^3^ this gives a theoretical removal capacity of 5781.97 mg L^−1^ (ppm) for these experiments. The 0.2 M solution contained a concentration of chromium (10 399 ppm) in excess to the removal capacity of the C3A added, and so total removal of chromium was not possible (the maximum removal capacity would be 55.6%). However, as 49.4% of the Cr present in the solutions was removed after 1 month, this shows that the method allows large amounts of Cr to be encapsulated in a solid phase. The experiments using lower concentrations (0.02 M and 0.01 M solutions) successfully removed most of the chromium (>99%). The 0.02 M experiment began with a solution Cr concentration of 1040 ppm and ended with 2.7 ppm after 1 week of reaction with C3A. The 0.01 M experiment started with a solution Cr concentration of 520 ppm and after only one day of reaction with C3A, this had reduced to 0.26 ppm. The initial Cr(vi) concentrations used in this study are high relative to waste water Cr(vi) concentrations, which can be up to 150 ppm.^10,11^ However, these results show that this method is extremely efficient at removing large amounts of chromium from solutions, which makes it promising to take forward for testing on real waste water. The current UK Environmental Quality Standard for Cr(vi) in freshwater is 0.034 ppm.^31^

The results from this study are comparable to previous studies on chromium removal from water using solidification or reduction methods. He and Suito (2002) achieved a reduction in Cr concentration from 50 ppm to 0.05 ppm (>99%)^32^ after 6 hours of shaking C3A and C12A7 (Ca_12_Al_14_O_33_) in a solution of potassium dichromate (K_2_Cr_2_O_7_). Other reduction/solidification studies, using different reagents, also achieve results comparable to this study, such as the 2020 study by Lu *et al.*, whereby collected groundwater samples were dosed with a mix of iron sulfate, sodium hydrosulfite and sodium metabisulfite. The chromium concentration of groundwater was reduced from 5.8 ppm to 0.4 ppm (93%),^33^ a lower removal efficiency than in our study. Graham *et al.* reduced Cr(vi) to Cr(iii) using calcium polysulfide^12^ and they found that using CaS_*x*_ removed >99.9% of the Cr(vi) from solutions with an initial Cr(vi) concentration of 1700 mg L^−1^. These results are comparable to our study.

### Analysis of the solid phase

#### Elemental analysis

**Fig. 2:**
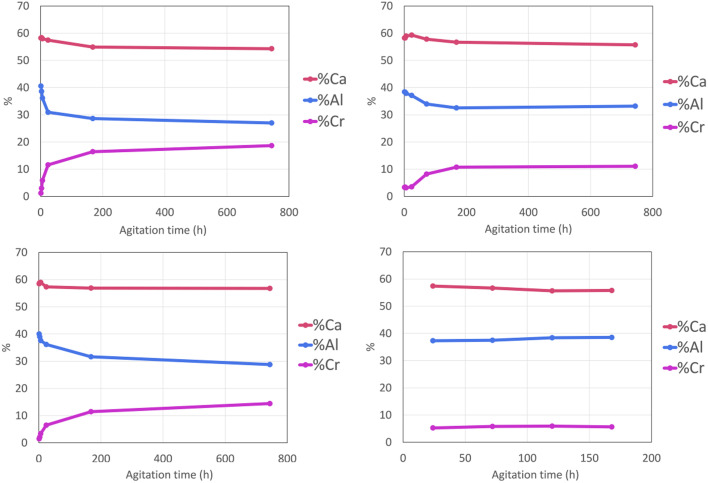
(a)–(d) ICP-OES results for the solid samples collected at various time points during the experiments (a) “0.2 M K_2_CrO_4_” (b) “0.1 M K_2_CrO_4_” (c) “0.02 M K_2_CrO_4_” and (d) “0.01 M K_2_CrO_4_” expressed as relative percentages (% Ca = red, % Al = blue, % Cr = pink).

Elemental analysis was also carried out on the solid samples using ICP-OES. Calcium, aluminium and chromium concentrations were measured using this technique. For each sample, the concentrations of these elements, in mol L^−1^, were measured and summed together to give the total Ca, Al, Cr content in the solid phase. Then a relative % content was calculated for each element using the following example formula:
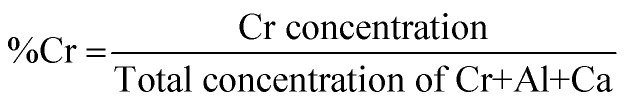


As such the results presented here do not give the absolute composition of the solid products but are indicative and can be used to confirm conclusions formed from other analysis methods. The relative percentages of Ca, Al and Cr are presented below (Fig. 2).

The elemental analysis clearly shows that the amount of chromium encapsulated in the solid phase increases as the reaction time increases in all solution concentrations except for the 0.01 M experiment which had already encapsulated the maximum amount of chromium after 24 hours of agitation. The relative calcium content stays approximately constant with the relative aluminium content decreasing as the chromium is removed from the solution into the solid phase. For the 0.2 M and 0.1 M experiments the measured chromium content increases with reaction time and the major uptake of chromium from the solutions into the solid phase occurs after 6 hours of reaction time. The 1 month sample, from the 0.2 M experiment, has a relative elemental composition of 54% calcium, 27% aluminium and 19% chromium which is almost equivalent to that expected from a pure sample of monochromate 8H_2_O, which has the ideal composition Ca_4_[Al(OH)_6_]_2_CrO_4_·8H_2_O, equivalent to relative percentages of 57% calcium, 29% aluminium, 14% chromium. For the 0.02 M and 0.01 M experiments the relative % Cr plateaus and stops increasing after a point (1 week reaction for 0.02 M and 1 day reaction time for 0.01 M). This suggests that these samples have incorporated and removed all of the available chromium in the solution. This assumption is further confirmed by the visual observation of the colour of the filtrate. The initial solutions of potassium chromate are vibrant yellow in colour, but the filtrates of the 1 week and 1 month samples for 0.02 M solutions and the 3, 5 and 7 day samples for 0.01 M solutions are colourless. This supports the results from the solution analysis, where the chromium concentration of the 0.02 M and 0.01 M solutions was determined to be close to detectable limits after these reaction times.

#### PXRD analysis

**Fig. 3:**
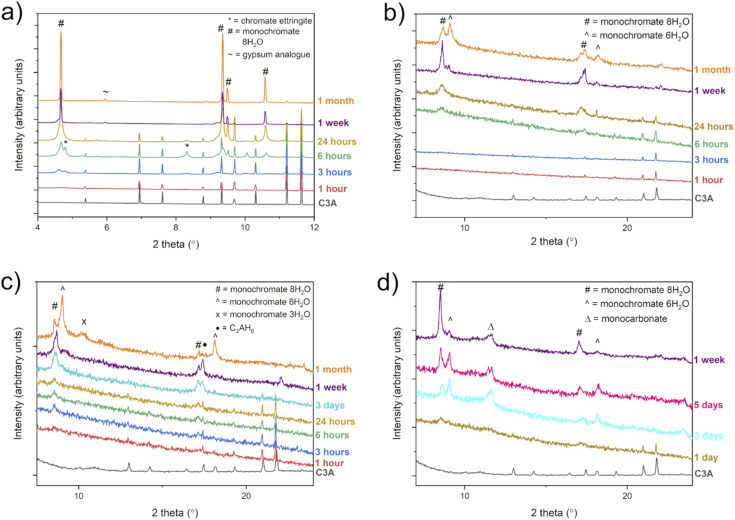
(a)–(d) Powder X-ray diffraction patterns of the solid samples collected at various time points during the experiments (a) “0.2 M K_2_CrO_4_, (b) “0.1 M K_2_CrO_4_”, (c) “0.02 M K_2_CrO_4_”, and (d) “0.01 M K_2_CrO_4_”. (a) Data collected on beamline I11 at the Diamond Light Source Synchrotron, *λ* = 0.82661 Å; (b)–(d) data collected on laboratory Bruker D8, *λ* = 1.5406 Å, transmission geometry. Reflections are labelled: * = chromate ettringite (Ca_6_[Al(OH)_6_]_2_(CrO_4_)_3_·26H_2_O), # = monochromate 8H_2_O (Ca_4_[Al(OH)_6_]_2_CrO_4_·8H_2_O), ^ = monochromate 6H_2_O (Ca_4_[Al(OH)_6_]_2_CrO_4_·6H_2_O), X = monochromate 3H_2_O (Ca_4_[Al(OH)_6_]_2_CrO_4_·3H_2_O), ∼ = gypsum analogue (Ca_2_CrO_4_·2H_2_O), • = C_3_AH_6_ (Ca_3_Al_2_(OH)_12_), and Δ = monocarbonate (Ca_4_[Al(OH)_6_]_2_CO_3_·5H_2_O).

The solid samples were analysed using PXRD, in order to identify the crystalline phases present at various times. These results (Fig. 3), along with those from FTIR analysis, can be used to confirm what phase(s) the chromium had been encapsulated in.

In the 0.2 M experiments (Fig. 3a), after 1 hour, unreacted C3A is the major phase present with only low intensity reflections at 4.6° 2*θ* (*d* spacing = 10.3 Å) and 4.8° 2*θ* (*d* spacing = 9.9 Å) observed in the PXRD data, which are assigned to monochromate 8H_2_O (Ca_4_[Al(OH)_6_]_2_CrO_4_·8H_2_O), # on Fig. 3a and chromate ettringite (Ca_6_[Al(OH)_6_]_2_(CrO_4_)_3_·26H_2_O), * on Fig. 3a. Monochromate can exist with various different hydration states (Ca_4_[Al(OH)_6_]_2_CrO_4_·*n*H_2_O, *n* = 3–8), distinguishable by the position of the (002) reflection in their PXRD pattern,^29^*e.g.* for 8H_2_O, (002) is observed at a *d* spacing of 10.3 Å. The only difference between these phases is the amount of interlayer water which causes the layer spacing reflection (002) (*d* spacing = 10.3 Å for 8H_2_O and *d* spacing = 9.7 Å for 6H_2_O) to shift. A decrease in the water content of the interlayer results in a contraction of the layers, reducing the *d* spacing of the layers and so the reflection is observed at a higher 2*θ* position on the diffraction pattern. The monochromate 8H_2_O and chromate ettringite reflections grow in intensity after 6 hours of reaction, however, after 24 hours there are no longer any reflections which can be assigned to chromate ettringite in the PXRD data. The samples reacted for 1 week and 1 month, show monochromate 8H_2_O as the main phase present, and there is no longer any evidence for the presence of the C3A starting material. A gypsum analogue phase (Ca_2_CrO_4_·2H_2_O) was also identified as present within these samples.

In the experiments with a lower solution concentration (0.1 M, 0.02 M, 0.01 M), chromate ettringite is not observed in any of the PXRD data and the chromium containing phases identified are monochromate (Ca_4_[Al(OH)_6_]_2_CrO_4_·*n*H_2_O, *n* = 3–8) phases. The reason that no ettringite is able to form in these lower concentration experiments is due to the stoichiometric differences between the ideal formulae of ettringite (Ca_6_[Al(OH)_6_]_2_(CrO_4_)_3_·26H_2_O) and of monochromate (Ca_4_[Al(OH)_6_]_2_CrO_4_·*n*H_2_O, *n* = 3–8). Ettringite requires a Ca : Cr ratio of 2 : 1, while monochromate requires a Ca : Cr ratio of 4 : 1. Therefore, it is more stoichiometrically favourable for monochromate to be formed when the amount of chromium in solution is decreased. This process occurs in cement systems, where ettringite will form from reaction of C3A and calcium sulfate hydrate (gypsum) until all the sulfate has been consumed, then the ettringite will react with more C3A to form monosulfate^34^ (eqn (1) and (2)). In the lower Cr concentration experiments, the lack of chromate means the monochromate is forming directly from the reaction of the C3A with the chromate in solution and no intermediate ettringite can be isolated.

1 Ca_3_Al_2_O_6_ + 3(CaSO_4_·2H_2_O) + 26H_2_O → Ca_6_[Al(OH)_6_]_2_(SO_4_)_3_·26H_2_O

2 Ca_6_[Al(OH)_6_]_2_(SO_4_)_3_·26H_2_O + 2(Ca_3_Al_2_O_6_) + 10H_2_O → 3(Ca_4_[Al(OH)_6_]_2_SO_4_·8H_2_O)

Eqn (1) Shows the formation of ettringite from reaction of C3A and gypsum, eqn (2) shows the subsequent reaction of ettringite with C3A to form a monosulfate phase.

Analysis of the solid phases using PXRD (Fig. 3b), from the experiments reacting C3A with 0.1 M K_2_CrO_4_ solutions, found that for reaction times of between 1 and 6 h, the main phase present was C3A, with little evidence of the formation of any further crystalline phases. After 6 h and 24 h, the 8H_2_O monochromate phase was identified (# on Fig. 3b), but the broad peaks suggest the phase is poorly crystalline. Analysis of the solid samples after reaction times of 1 week and 1 month, identified the presence of two monochromate phases with different hydration levels, 8H_2_O (#) and 6H_2_O (^).

When the concentration of the solutions is lowered further to 0.02 M and 0.01 M, analysis of the solid phases from these experiments showed the presence of monochromate phases with different hydration levels as the main products but there are also phases identified that contain no chromium. For the 0.02 M experiment (Fig. 3c) C_3_AH_6_ (Ca_3_Al_2_(OH)_12_) is identified as present. This is the hydrated form of the starting material C3A and can be identified by its (112) reflection at 17.3° 2*θ* (represented by • in Fig. 3c). C_3_AH_6_ was identified as present in all PXRD patterns with a reaction time of more than 6 hours. With the C3A now in excess (∼2 : 1 molar ratio C3A : Cr) the excess C3A has hydrated as there are not enough available chromate ions in solution to react with. A similar reaction occurs in the 0.01 M experiment (Fig. 3d) where the excess C3A (now ∼4 : 1 molar ratio C3A : Cr) is not simply hydrating but reacting with carbonate absorbed from the air in the reaction vessel and forming monocarbonate (Ca_4_[Al(OH)_6_]_2_CO_3_·5H_2_O). Monocarbonate was identified in samples with a reaction time of 3 days or greater by the presence of its (011) layer reflection at 11.5° 2*θ* (represented by ^ in Fig. 3d) and its presence can be confirmed using FTIR (see next Section). Monocarbonate has a layered crystal structure similar to the monochromate phases but with carbonate ions between the calcium aluminate layers and a smaller interlayer distance. There is also the possibility that minor impurity phases may be present in quantities too small (*i.e.* <3 wt%) to be detected by laboratory PXRD techniques. The laboratory diffractometer has a lower intensity, flux and resolution than the synchrotron beamline used for the 0.2 M samples.

An important factor in this solid state characterisation is the solution pH, which has an effect on chromium speciation. Cr(vi) exists as CrO_4_^2−^ at alkaline pH and as Cr_2_O_7_^2−^ at lower acidic pH. The pH of the initial K_2_CrO_4_ solutions were ∼9 and on addition of C3A this rose to above 12. Therefore, Cr(vi) must exist as the polyanion CrO_4_^2−^ in these solutions at all reaction times. This high alkaline pH is beneficial for encapsulation into cement minerals but can also affect which products are precipitated. Geelhoed *et al.* (2002) studied the leaching of Cr(vi) from COPR at various pH conditions.^35^ They found that at pH values of 11 and above (which is within the working pH range of our study) the two main phases which Cr(vi) was found to be present in were Cr(vi)-substituted hydrogarnet (Ca_3_Al_2_(H_4_O_4_, CrO_4_)_3_) and monochromate (Ca_4_[Al(OH)_6_]_2_CrO_4_·6H_2_O). At pH 9.5–11 they found that chromate ettringite (Ca_6_[Al(OH)_6_]_2_(CrO_4_)_3_·26H_2_O) was the main chromium containing phase, formed as a result of monochromate dissolving at lower pH. As our study was carried out at pH >11 and we have shown monochromate is the main Cr(vi)-containing phase, our results are in agreement with Geelhoed's. The pH of our solutions was not controlled, and any rise in solution pH was as a result of the addition of C3A. In the future, the effect of altering the pH could be investigated in order to target the production of chromate ettringite. Understanding what phases are produced under different experimental conditions is vital in order to validate whether a material will successfully remove PTEs in real waste water. This is one of the reasons that solid state studies are vital for developing new remediation materials and methods.

#### FTIR analysis

**Fig. 4:**
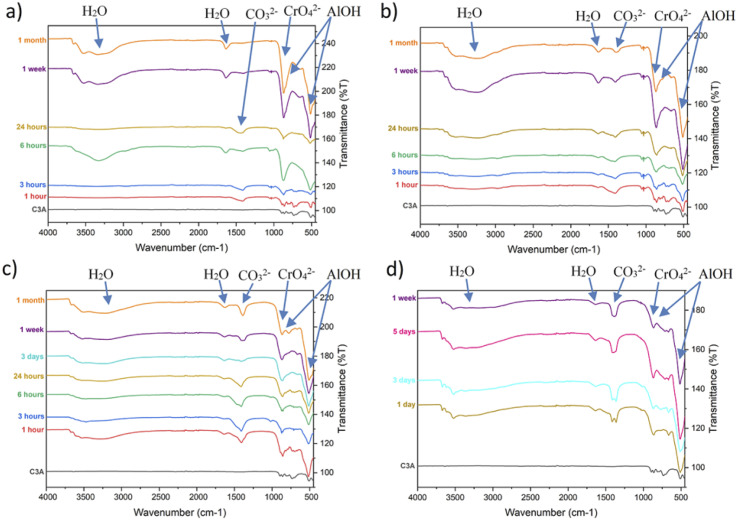
(a)–(d) FTIR spectra of the solid samples collected at various time points during the experiments (a) “0.2 M K2CrO4”, (b) “0.1 M K2CrO4”, (c) “0.02 M K2CrO4”, and (d) “0.01 M K2CrO4” with bands for key functional groups indicated by arrows and labels.

FTIR spectra were also recorded on these samples (Fig. 4) and these data can be used in conjunction with the PXRD data to confirm the phases identified as present in the solid samples.

Analysis of the solid phases using FTIR (Fig. 4a) from the experiments where C3A was reacted with 0.2 M K_2_CrO_4_, found that the spectra for all samples with a reaction time of longer than 3 hours contain the band for chromate; this supports the PXRD data and shows that chromate has been removed from solution and is now in the solid phase. However, the chromate band (863 cm^−1^)^36^ and one of the bands for AlOH (856 cm^−1^)^37^ overlap which makes identification more difficult. The chromate band is stronger than the AlOH and so AlOH is observed as a shoulder at slightly lower wavenumber on the chromate peak. Carbonate stretches are observed in all the spectra up to a reaction time of 1 week, suggesting the presence of a carbonate containing phase, which is not detected by PXRD, possibly due to its presence in small quantities, lower than the detection limit (∼3 wt%). Monocarbonate (Ca_4_[Al(OH)_6_]_2_CO_3_·5H_2_O) and hemicarbonate (Ca_4_Al_2_(OH)_13_(CO_3_)_0.5_.5.5H_2_O) phases, with similar layered structures to the expected monochromate, are known to exist in cement systems where there has been exposure to air.^38^ In these layered “mono” phases the chromate and carbonate are positioned between the calcium alumino layers and therefore can easily exchange with other ions. It is suggested that a carbonate containing precursor (monocarbonate/hemicarbonate) is forming and then chromate ions exchange into the structure forming the monochromate 8H_2_O final product. As the carbonate stretch is not present in the IR spectrum for the 1 month sample, it can be deduced that the excess of chromate in the solution has exchanged with and replaced any carbonate that had initially been absorbed from the air into the mono phase. Analysis of the data collected from the experiments where C3A reacted with 0.1 M (Fig. 4b) and 0.02 M (Fig. 4c), found that the spectra contain the bands for chromate and carbonate. Analysis of the PXRD data collected on the 1 hour samples (and the 3 hour sample for 0.1 M) do not show any crystalline chromate-containing phases. This suggests that carbonate and chromate may be absorbing onto the surface of the C3A, forming amorphous phases initially, or the amount of chromate-containing phases present is less than that detectable by these PXRD methods (∼3–5 wt%). The carbonate bands are significant for all samples, and this indicates that the monochromate phases identified have some carbonate also incorporated into the structure through absorption from the air. The carbonate stretch is still present in the spectra for the final samples, collected after 1 month of reaction. This is because there is an insufficient amount of chromate ions in these solutions and as such, they cannot replace the carbonate to produce any pure monochromate phases.

Significant carbonate bands are found in the FTIR spectra for experiments where C3A was reacted, for more than 1 day, with 0.01 M K_2_CrO_4_ (Fig. 4d). These can be attributed to the presence of a monocarbonate phase, identified by PXRD. However, the carbonate band is also prominent in the 1 day sample, but the PXRD pattern for this sample did not show the presence of any strong reflections that could be assigned to a monocarbonate phase. This suggests that monocarbonate formation had already begun at short times, but the product was either poorly crystalline, amorphous or present in quantities less than that detectable by these PXRD methods (∼3–5 wt%). There is evidence that calcite and vaterite (polymorphs of CaCO_3_) form from solution *via* Amorphous Calcium Carbonates (ACC), where an amorphous CaCO_3_ precursor forms initially.^39,40^ As such, amorphous carbonate precursor formation is possible in this experiment and would explain the presence of carbonate stretches in the FTIR spectra. Overall, these results show that the absorption of carbonate is a major contributor to the products forming in these experiments where the chromium concentration is lower. As real wastewater may have a relatively low concentration of chromium, compared to the concentrations investigated in this study, the absorption of carbonate should be considered in any future experiments to remediate chromium using these materials. The high pH of these solutions (>12) could also be affecting the carbonate absorption so future studies that control the pH should pay careful attention to the presence of carbonate bands in FTIR spectra.

**Fig. 5:**
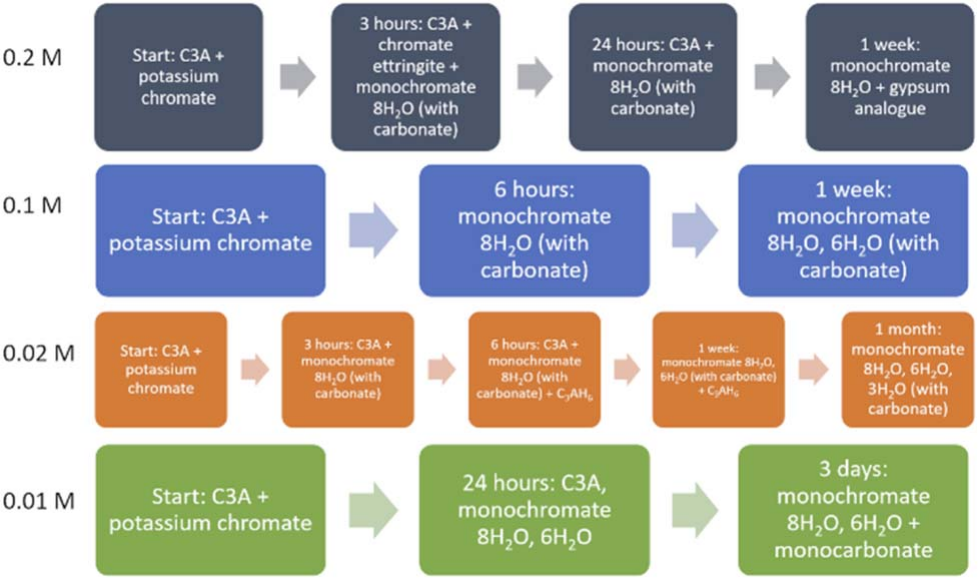
Reaction pathways for the reaction of C3A with various concentrations of solutions of K_2_CrO_4_ (note: “with carbonate” refers to the presence of CO_3_^2−^ groups being intercalated into the layered structures).

A summary diagram of the reaction pathways for each experiment with a different solution concentration, determined using a combination of all the results obtained in this study, is shown in Fig. 5.

The determination of reaction pathways for the hydration of C3A in solutions of differing chromium concentrations is important as it helps our understanding of how certain phases form and the solid–liquid interactions that may occur in cement materials.

**Fig. 6:**
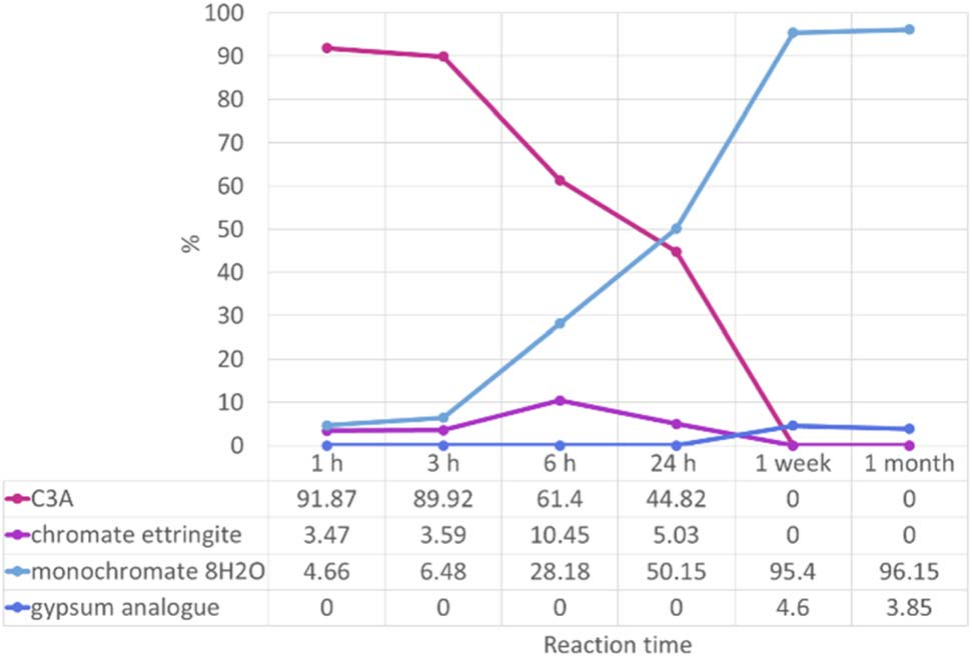
Proportions, as a percentage, of phases identified as present by quantitative phase analysis by Rietveld refinement of “0.2 M K_2_CrO_4_” PXRD data.

The high-resolution PXRD data collected on the 0.2 M experiment samples allowed quantitative phase analysis (QPA) to be carried out. This was carried out by structural refinement of the PXRD data using the Rietveld refinement method.^41,42^ This method uses structural models for the identified phases to calculate PXRD patterns, which are matched to the observed data and differences minimised through a least squares process. The fitting of multiple phases to observed data allows the proportions of the phases to be calculated. The results of the QPA are shown in Fig. 6.

Results from QPA show the approximate numerical proportions of phases in the solid products from the 0.2 M experiment. This supplements the information we can obtain from phase identification alone (summarised in Fig. 5), as now it is confirmed that the monochromate 8H_2_O phase is the dominant product of the reaction between C3A and a 0.2 M solution of potassium chromate. Chromate ettringite only makes up a small percentage (up to ∼10%) of the products at any reaction time. It is important to know the composition of the reaction products as their stability will need to be investigated in the future in order to determine the risk of the encapsulated pollutant ion being released.

## Conclusions

Our studies into the hydration of C3A in the presence of hexavalent chromium have found that chromium was successfully removed from solution and encapsulated in a solid phase. These experiments showed a removal of >99% of the chromium from solution which is comparable to other current chromium remediation methods and studies and provides proof of concept for using C3A as a chromium remediation material. Differences that occur when lower concentration chromium solutions are used have been identified. All solid and liquid products were fully characterised and monochromate phases were found to be the dominant Cr(vi) containing solid products. Carbonate was found to easily incorporate into the solid products of these reactions, with this effect being more pronounced at lower Cr solution concentrations. These are all important factors to consider when further developing this as a waste remediation process. In the future, this remediation process could eventually be implemented into polluted aqueous waste streams, in order to remediate the waste. However, in the future, there needs to be further carefully controlled lab studies, where the solutions have greater complexity, to assess the impact of competing ions which may be present in water samples collected from polluted environments. In particular, the effect of competing sulfate should be investigated to determine if it will preferentially react to form the cement minerals ettringite and monosulfate or solid solutions which can form containing both sulfate and chromate anions in both ettringite-Cr-ettringite^43^ and monosulfate–monochromate systems.^28^ The relative stabilities of chromate ettringite and monochromate also needs to be investigated in the future, to ensure that any encapsulated chromium is unable to leach back out of the solid products.

As we have identified the reaction pathways for C3A hydrating in Cr(vi) containing solutions and can predict what phases will form, the work presented here could have real consequences for water treatment. It shows that using this simple method, requiring only one low-cost starting material, can effectively remove large quantities of potentially toxic elements in short periods of time. The starting material, tricalcium aluminate, is non-hazardous, and the products of the reaction are known phases which have been fully characterised by this study. Encapsulating waste ions in known materials, which are products of cement hydration/formation, means that there is also the potential for the waste to be stabilised fully in a cement matrix, which would allow long term stabilisation and pose no further threat to the environment through leaching or reduction.

## Author contributions

Rebecca Rae: Conceptualisation, project administration, formal analysis, investigation, methodology, writing-original draft. Margaret C. Graham: Writing-review and editing. Caroline A. Kirk: Conceptualisation, project administration, resources, supervision, writing-review and editing.

## Conflicts of interest

There are no conflicts of interest to declare.

## Supplementary Material

RA-012-D2RA04497H-s001

## References

[cit1] WWAP (United Nations World Water Assessment Programme) , The United Nations world water development report, 2017: Wastewater: the untapped resource, Paris, 2017

[cit2] UNEP FI (United Nations Environment Programme Finance Initiative) , Half Full or Half Empty? A Set of Indicative Guidelines for Water-Related Risks and an Overview of Emerging Opportunities for Financial Institutions, Geneva, 2007, vol. 29

[cit3] GWI (Global Water Intelligence) , Industrial Water Technology Markets 2015, Oxford, 2015

[cit4] Salnikow K., Zhitkovich A. (2008). Chem. Res. Toxicol..

[cit5] Zhitkovich A. (2011). Chem. Res. Toxicol..

[cit6] Pellerin C., Booker S. M. (2000). Environ. Health Perspect..

[cit7] Rai D., Eary L. E., Zachara J. M. (1989). Sci. Total Environ..

[cit8] Tumolo M., Ancona V., De Paola D., Losacco D., Campanale C., Massarelli C., Uricchio V. F. (2020). Int. J. Environ. Res. Public Health.

[cit9] Broadway A., Cave M. R., Wragg J., Fordyce F. M., Bewley R. J. F., Graham M. C., Ngwenya B. T., Farmer J. G. (2010). Sci. Total Environ..

[cit10] Whalley C., Hursthouse A., Rowlatt S., Iqbal-Zahid P., Vaughan H., Durant R. (1999). Water, Air, Soil Pollut..

[cit11] Farmer J. G., Thomas R. P., Graham M. C., Geelhoed J. S., Lumsdon D. G., Paterson E. (2002). J. Environ. Monit..

[cit12] Graham M. C., Farmer J. G., Anderson P., Paterson E., Hillier S., Lumsdon D. G., Bewley R. J. F. (2006). Sci. Total Environ..

[cit13] Sim G. , Graham M. C., Ngwenya B. T. and Fordyce F. M., Understanding chromium behaviour in copr-impacted sediments in the polmadie burn, Glasgow, 4th IIES Workshop, Edinburgh 1–3 July 2018, 2018

[cit14] Bearcock J. M., Smedley P. L., Fordyce F. M., Everett P. A., Ander E. L. (2017). Earth Environ. Sci. Trans. R. Soc. Edinburgh.

[cit15] James B. R. (2001). Environ. Geochem. Health.

[cit16] Bewley R. J. F., Jeffries R., Watson S., Granger D. (2001). Environ. Geochem. Health.

[cit17] Geelhoed J. S., Meeussen J. C. L., Roe M. J., Hillier S., Thomas R. P., Farmer J. G., Paterson E. (2003). Environ. Sci. Technol..

[cit18] Farmer J. G., Paterson E., Bewley R. J. F., Geelhoed J. S., Hillier S., Meeussen J. C. L., Lumsdon D. G., Thomas R. P., Graham M. C. (2006). Sci. Total Environ..

[cit19] Geioushy R. A., El-Sheikh S. M., Azzam A. B., Salah B. A., El-Dars F. M. (2020). J. Hazard. Mater..

[cit20] El-Sheikh S. M., Azzam A. B., Geioushy R. A., El Dars F. M., Salah B. A. (2021). J. Alloys Compd..

[cit21] Koťátková J., Zatloukal J., Reiterman P., Kolář K. (2017). J. Environ. Radioact..

[cit22] Gougar M. L. D., Scheetz B. E., Roy D. M. (1996). Waste Manage..

[cit23] Mondal P., Jeffery J. W. (1975). Acta Crystallogr., Sect. B: Struct. Crystallogr. Cryst. Chem..

[cit24] Bailey J. E., Hampson C. J., Bensted J. (1983). Philos. Trans. R. Soc., A.

[cit25] Hampson C. J., Bailey J. E. (1983). J. Mater. Sci..

[cit26] Christensen A. N., Jensen T. R., Hanson J. C. (2004). J. Solid State Chem..

[cit27] Leisinger S. M., Lothenbach B., Le Saout G., Kägi R., Wehrli B., Johnson C. A. (2010). Environ. Sci. Technol..

[cit28] Leisinger S. M., Lothenbach B., Le Saout G., Johnson C. A. (2012). Cem. Concr. Res..

[cit29] Göske J., König U., Pöllmann H. (2004). Mater. Sci. Forum.

[cit30] Thompson S. P., Parker J. E., Potter J., Hill T. P., Birt A., Cobb T. M., Yuan F., Tang C. C. (2009). Rev. Sci. Instrum..

[cit31] SEPA , Supporting Guidance (WAT-SG-53) Environmental Quality Standards and Standards for Discharges to Surface Waters, 2020

[cit32] He H., Suito H. (2002). ISIJ Int..

[cit33] Lu S.-F., Wu Y.-L., Chen Z., Li T., Shen C., Xuan L.-K., Xu L. (2020). Environ. Sci. Pollut. Res..

[cit34] Taylor H. F. W. , Cement chemistry 2nd edn, Thomas Telford, London, 2nd edn, 1997

[cit35] Geelhoed J. S., Meeussen J. C. L., Hillier S., Lumsdon D. G., Thomas R. P., Farmer J. G., Paterson E. (2002). Geochim. Cosmochim. Acta.

[cit36] Malchos M., Jansen M. (1998). Z. Naturforsch., B: Anorg. Chem., Org. Chem..

[cit37] Yuke L., Zhengmao Y., Shuxian W., Shuxin L., Cheng X. (2019). J. Therm. Anal. Calorim..

[cit38] Kuzel H. J. (1996). Cem. Concr. Compos..

[cit39] Gebauer D., Gunawidjaja P. N., Ko J. Y. P., Bacsik Z., Aziz B., Liu L., Hu Y., Bergström L., Tai C. W., Sham T. K., Edén M., Hedin N. (2010). Angew. Chem., Int. Ed..

[cit40] Lam R. S. K., Charnock J. M., Lennie A., Meldrum F. C. (2007). CrystEngComm.

[cit41] Rietveld H. M. (1967). Acta Crystallogr..

[cit42] Rietveld H. M. (1969). J. Appl. Crystallogr..

[cit43] Rae R. , PhD Thesis, University of Edinburgh, 2021, (also paper in preparation)

