# Supplementary material for: Investigating the hydration of C3A in the presence of the potentially toxic element chromium–a route to remediation?
Source: RSC Adv. 2022 Oct 12;12(45):29329–37. doi: 10.1039/d2ra04497h (PMC9555287; doi:10.1039/d2ra04497h)

Electronic Supplementary Material (ESI) for RSC Advances.
This journal is © The Royal Society of Chemistry 2022

Supplementary Information

Figure A: Structural diagram of part of the structure of C3A (tricalcium aluminate, $Ca_3Al_2O_6$) illustrating the coordination of calcium (blue) to oxygen (green), and aluminium (red) to oxygen (Figure produced using the crystal structure drawing package VESTA (Copyright (C) 2006-2018, Koichi Momma and Fujio Izumi))

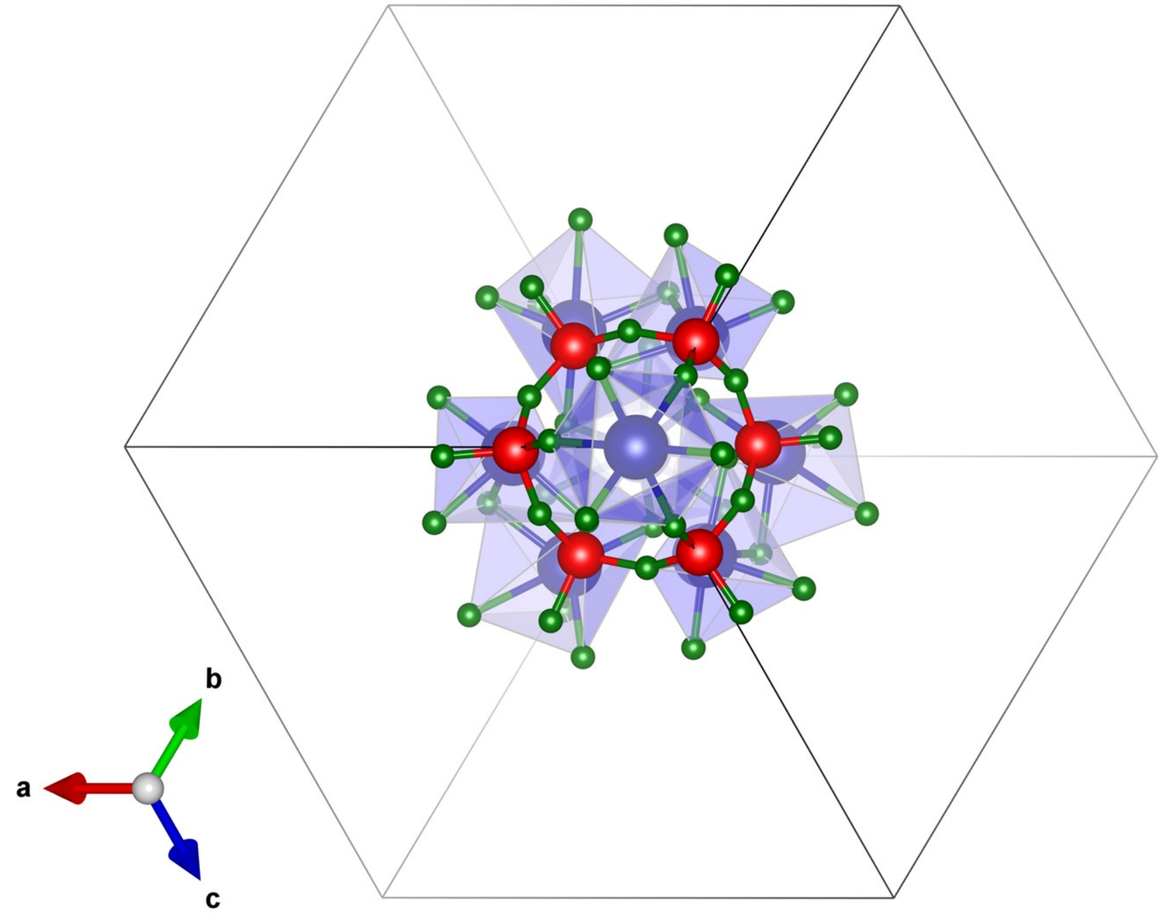

Supplement: RA-012-D2RA04497H-s001 [file RA-012-D2RA04497H-s001.pdf]
